# Analysis of Anti-Influenza Virus Neuraminidase Antibodies in Children, Adults, and the Elderly by ELISA and Enzyme Inhibition: Evidence for Original Antigenic Sin

**DOI:** 10.1128/mBio.02281-16

**Published:** 2017-03-21

**Authors:** Madhusudan Rajendran, Raffael Nachbagauer, Megan E. Ermler, Paul Bunduc, Fatima Amanat, Ruvim Izikson, Manon Cox, Peter Palese, Maryna Eichelberger, Florian Krammer

**Affiliations:** a Department of Microbiology, Icahn School of Medicine at Mount Sinai, New York, New York, USA; b Protein Sciences Corp., Meriden, Connecticut, USA; c Department of Medicine, Icahn School of Medicine at Mount Sinai, New York, New York, USA; d Division of Viral Products, Center for Biologics Evaluation and Research, Food and Drug Administration, Silver Spring, Maryland, USA; CDC; Emory University

**Keywords:** influenza, influenza virus, neuraminidase, neuraminidase inhibition

## Abstract

Antibody responses to influenza virus hemagglutinin provide protection against infection and are well studied. Less is known about the human antibody responses to the second surface glycoprotein, neuraminidase. Here, we assessed human antibody reactivity to a panel of N1, N2, and influenza B virus neuraminidases in different age groups, including children, adults, and the elderly. Using enzyme-linked immunosorbent assays (ELISA), we determined the breadth, magnitude, and isotype distribution of neuraminidase antibody responses to historic, current, and avian strains, as well as to recent isolates to which these individuals have not been exposed. It appears that antibody levels against N1 neuraminidases were lower than those against N2 or B neuraminidases. The anti-neuraminidase antibody levels increased with age and were, in general, highest against strains that circulated during the childhood of the tested individuals, providing evidence for “original antigenic sin.” Titers measured by ELISA correlated well with titers measured by the neuraminidase inhibition assays. However, in the case of the 2009 pandemic H1N1 virus, we found evidence of interference from antibodies binding to the conserved stalk domain of the hemagglutinin. In conclusion, we found that antibodies against the neuraminidase differ in magnitude and breadth between subtypes and age groups in the human population. (This study has been registered at ClinicalTrials.gov under registration no. NCT00336453, NCT00539981, and NCT00395174.)

## IMPORTANCE

Anti-neuraminidase antibodies can afford broad protection from influenza virus infection in animal models and humans. However, little is known about the breadth and magnitude of the anti-neuraminidase response in the human population. Here we assessed antibody levels of children, adults, and the elderly against a panel of N1, N2, and type B influenza virus neuraminidases. We demonstrated that antibody levels measured by ELISA correlate well with functional neuraminidase inhibition titers. This is an important finding since ELISA is a simpler method than functional assays and can be implemented in high-throughput settings to analyze large numbers of samples. Furthermore, we showed that low titers of broadly cross-reactive antibodies against neuraminidase are prevalent in humans. By the use of an appropriate vaccination strategy, these titers could potentially be boosted to levels that might provide broad protection from influenza virus infection.

## INTRODUCTION

Influenza virus infections remain a major public health concern worldwide. The available influenza virus vaccines represent valuable tools to prevent infection when the vaccine strains are well matched with the circulating strains (1). Immune responses induced by available inactivated influenza virus vaccines primarily target the immunodominant globular head domain of the hemagglutinin (HA) which is responsible for viral attachment and fusion. These antibodies (Abs) can be strongly neutralizing, but their target, the globular head domain, has inherently high plasticity (2). The virus has the ability to escape the immune response by introducing mutations into this domain, a phenomenon called antigenic drift.

The second major surface glycoprotein, the neuraminidase (NA), is often overlooked as a vaccine antigen (3, 4). The NA has enzymatic sialidase activity which is important for the transport of incoming virions through mucins and for the release of budding virus from infected cells (3). Inhibition of this sialidase activity is a major mechanism of action of NA-directed antibodies, but other mechanisms which involve antibody-effector cell interactions might be important as well. Inactivated influenza virus vaccines usually induce low immune responses to the NA (5–7), likely due to the immunosubdominance of the NA when it is presented to the immune system in conjunction with the HA (5, 8–10). In addition, NA tetramers are outnumbered on the influenza virus particles by HA spikes 1:4 (11). However, antibodies against the NA can protect from influenza virus infection in animal models (6, 12–23). In fact, anti-NA titers have been shown to confer protection from influenza virus infection and disease in humans as well (7, 8, 14). A recent human challenge study performed at the U.S. National Institutes of Health revealed that neuraminidase inhibition (NI) titers correlated well with protection against pandemic H1N1 disease symptoms and duration of shedding (24).

Here we describe the breadth, functionality, and subtypes/isotypes of anti-NA antibodies in children, adults, and the elderly. In addition, we explore the correlation between binding antibody titers measured in an enzyme-linked immunosorbent assays (ELISA) and levels of functional NI antibodies measured in an enzyme-linked lectin assay (ELLA) (25, 26).

## RESULTS

### Breadth, age dependence, and subtype/isotype of anti-NA titers.

**FIG 1:**
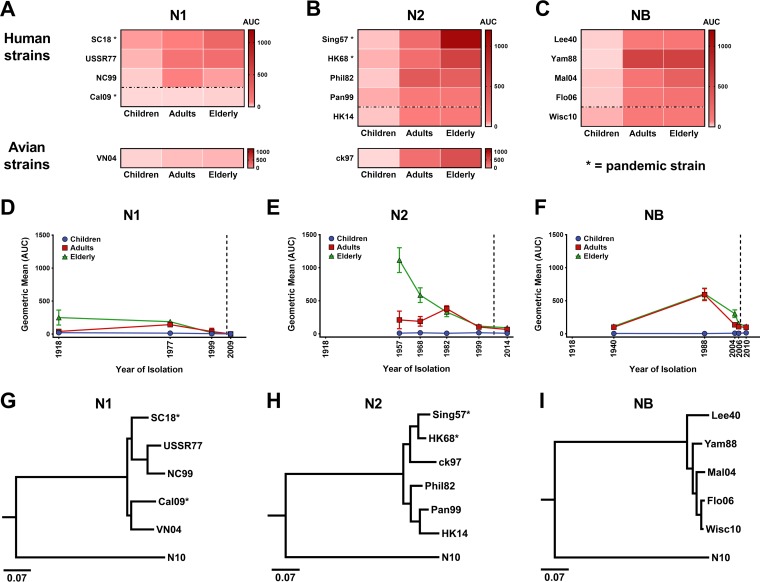
Anti-NA titers against human and avian influenza virus isolates in serum of children (6 to 59 months of age), adults (18 to 49 years of age), and the elderly (≥65 years of age). (A to C) Mean baseline anti-NA ELISA titers of the three different age groups were visualized using heat maps for (A) N1 (SC18, USSR77, NC99, Cal09, and VN04), (B) N2 (Sing57, HK68, Phil82, Pan99, and ck97), and (C) influenza B virus NA (NB; Lee40, Yam88, Mal04, Flor06, and Wisc10). *, pandemic strain. (D to F) Serum reactivity profiles of the three age cohorts against (D) N1, (E) N2, and (F) influenza B virus NA spanning the years 1918 to 2014. Serum reactivity profiles are shown using geometric mean titers, with error bars displaying standard errors of the means. The dashed lines correspond to 2006/2008, indicating the years during which the serum samples were collected. AUC, area under the curve. (G to I) Phylogenetic trees of influenza virus neuraminidase proteins used in this study: (G) N1; (H) N2; (I) influenza B virus NA (NB). Scale bars represent a 7% difference in amino acid identity. The trees depicted in panels G to I were rooted using the sequence of the N10 NA-like protein (A/little yellow-shouldered bat/Guatemala/060/2010).

The breadth of anti-NA antibody titers in different age groups has not been extensively studied. Here, we used a panel of purified recombinant N1, N2, and influenza B virus NAs in a quantitative ELISA to measure antibody titers in children (6 to 59 months of age), adults (18 to 49 years of age), and the elderly (65 years of age and older). Sera were collected as part of clinical trials conducted with the influenza virus vaccine Flublok between 2006 and 2008, before the 2009 pandemic H1N1 strain began to circulate. NA titers (day 0) were visualized as heat maps and reactivity profiles (Fig. 1). Titers against N1 were generally low, with the strongest reactivity to the A/South Carolina/1/1918 (SC18, H1N1) N1 in the elderly and the A/USSR/92/1977 (USSR77, H1N1) N1 in the adults (Fig. 1A and D). The reactivity to a contemporary N1 from A/New Caledonia/20/1999 (NC99, H1N1) was lower, including in the youngest cohort. Furthermore, reactivity to the N1 of the pandemic A/California/04/2009 (Cal09, H1N1) virus, to which these individuals had not been exposed, was barely detectible. We also tested reactivity to the avian N1 of the A/Vietnam/1203/2004 (VN04, H5N1) strain and found low but detectible titers in the adults and elderly. Interestingly, the reactivity to the VN04 N1 was even slightly higher than against the Cal09 N1 for both groups. N2 titers were in general much higher than N1 titers (Fig. 1B and E). Again, the elderly had the strongest reactivity, with the highest titers against the pandemic A/Singapore/1/1957 (Sing57, H2N2) N2 and lower titers against more-modern isolates, suggesting a recall response to the earlier antigen, referred to as “antigenic sin” or back-boosting (27–29). Adults had a relatively high titer against the N2 from A/Philippines/2/1982 (Phil82, H3N2), with lower titers against older or more-recent isolates, suggesting that the first virus that they had encountered in their life had left an immunological imprint. Children had consistently low titers against all isolates. Interestingly, cross-reactivity to an N2 from a future (i.e., subsequently encountered) isolate, A/Hong Kong/4801/2014 (HK14, H3N2), was relatively high in both adults and the elderly and reactivity to an N2 from an avian isolate (A/chicken/Hong Kong/G9/1997 [ck97, H9N2]) was even higher, suggesting the presence of conserved epitopes. Titers against influenza B virus NAs were relatively high in adults and the elderly, with a distinct peak for the NA from the B/Yamagata/16/1988 (Yam88) virus and lower titers for B/Lee/1940 (Lee40) and more-recent isolates (Fig. 1C and F). Reactivity to a future isolate, B/Wisconsin/1/2010 (Wisc10), was relatively high, again suggesting the presence of conserved epitopes. Serum of children reacted poorly to the influenza B virus NAs.

We also examined the antibody subtype (see Fig. S1A to C in the supplemental material) and isotype (Fig. S1D to F) distribution of anti-N1, anti-N2, and anti-influenza B virus NA antibodies. In general, the pattern reflected the distribution seen with antibodies to HA (30–32), with the exception of elevated IgG4 titers against NC99 N1 and elevated IgG2 titers against Yam88 NB (Fig. S1). In summary, titers against N1 appeared to be lower than titers against N2 or influenza B virus NA; titers increased with age, and they were in general higher against strains that had circulated during the childhood/youth of the individuals in the adult and elderly cohorts.

10.1128/mBio.02281-16.1 FIG S1  Isotype and IgG subtype composition of the anti-NA response of the sera from three different age groups. (A to C) Mean IgG1, IgG2, IgG3, and IgG4 antibody reactivity to (A) NC99 N1, (B) Pan99 N2, and (C) Yam88 NB. (D to F) Mean IgA and IgM antibody response to (D) New Caledonia 1999 N1, (E) Panama 1999 N2, and (F) Yam88 influenza B virus NA. Download FIG S1, TIF file, 0.7 MB. Copyright © 2017 Rajendran et al. 2017 Rajendran et al. This content is distributed under the terms of the Creative Commons Attribution 4.0 International license.

### Correlation between binding and functionality of anti-NA antibodies.

While binding of antibodies to the NA protein as assessed by ELISA is an indication of the presence of an immune response, it does not necessarily indicate that this immune response is functional and/or protective. Therefore, we measured functional antibody titers against four representative NAs using an enzyme-linked lectin assay (ELLA) to determine neuraminidase inhibition (NI) titers. These NAs included N1 from NC99, N2 from A/Panama/2007/1999 (Pan99, H3N2), influenza B virus NA from Yam88, and N1 NA from Cal09 virus (as an example of a future strain that these individuals had not been previously exposed to). To minimize anti-HA head antibody-based interference (25, 33), state-of-the-art NI assays using H6NX reassortants were employed. Humans have little or no H6 head-specific preexisting immunity; therefore, reassortant viruses that contain an H6 HA in combination with the NA of interest give a readout that is more reflective of the true NA-based NI titer (25).

**FIG 2:**
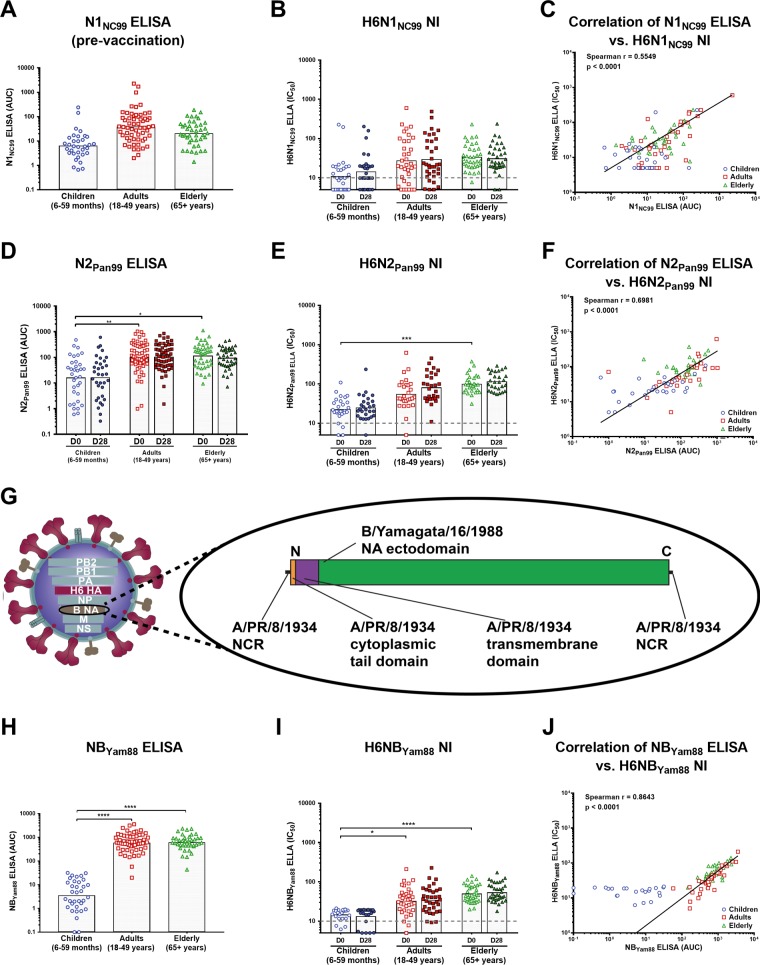
Relationship between binding and functionality of antibodies against prepandemic seasonal N1, N2, and type B influenza virus NA. (A and B) Geometric mean titers for children, adults, and the elderly against recombinant NC99 N1 as measured by ELISA (prevaccination, shown as the area under the curve [AUC]) (A) and ELLA titers (pre- and postvaccination, shown as IC_50_ values) against H6N1_NC99_ virus (B). The dashed line indicates the starting serum dilutions used in the ELLA assays. (C) Correlation between NC99 N1 ELLA and ELISA titers (Spearman *r* = 0.5549, *P* < 0.0001). (D and E) The same analyses were also performed for anti-N2 antibodies with recombinant Pan99 N2 as an ELISA substrate (pre- and postvaccination) (D) and H6N2_Pan99_ as an ELLA reagent (pre- and postvaccination) (E). D, day. (F) Similarly to the data determined with N1, the binding and functional titers against Pan99 N2 correlated well (Spearman *r* = 0.6981, *P* < 0.0001). (G) Design of the H6NB_Yam88_ virus that was rescued specifically for this study. The virus is a reassortant virus that combines the internal genes of H1N1 virus A/PR/8/34, an H6 HA, and a chimeric NA. The NA consists of the ectodomain of the B/Yamagata/16/1988 NA combined with the cytoplasmic tail and transmembrane domains from the A/PR/8/34 N1 NA. The open reading frame is flanked by 5′ and 3′ noncoding regions (NCRs) of the A/PR/8/34 NA. (H and I) Geometric mean titers for children, adults, and the elderly against recombinant Yam88 NB as measured by ELISA (prevaccination) (H); ELLA titers (pre- and postvaccination) against the novel H6NB_Yam88_ virus (I). (J) Relationship between binding and functional Yam88 NA titers (Spearman *r* = 0.8643, *P* < 0.0001).

As mentioned above, ELISA titers to NC99 N1 were low in children, highest in the adults, and slightly lower again in the elderly (Fig. 1A and Fig. 2A). NI titers measured in the ELLA using the H6N1_NC99_ virus reflected the results obtained by N1 ELISA. Children had the lowest titers, and titers of the adults and elderly were higher (Fig. 2B). No increase in ELISA or NI geometric mean titers from the pre-recombinant HA vaccination time point to the post-recombinant HA vaccination time point was detected (Fig. 2A and B), and we found good correlation between NI and ELISA titers, with a Spearman *r* value of 0.5549 (Fig. 2C).

ELISA titers for N2 NA from Pan99 were relatively low in children and higher in the older age groups. Here, we also measured ELISA titers of postvaccination sera and found no increase (as expected since the vaccine did not contain NA) (Fig. 2D). We then conducted an ELLA assay using an H6N2_Pan99_ virus. The measured titers followed the ELISA titers, with low levels for NI in children and higher levels for NI in the adults and elderly (Fig. 2E). Again, we performed an analysis and found that the ELISA titers against N2 significantly correlated with the H6N2_Pan99_ ELLA titers (*r* = 0.6981, Fig. 2F).

As described above and shown in Fig. 1, ELISA titers against the Yam88 influenza B virus NA were low in children but relatively high in the adults and the elderly (Fig. 2H). We also assessed the NI titers with an H6 reassortant virus. For the purpose of this study, we rescued a novel H6 virus that expresses the NA ectodomain of the B/Yamagata/16/88 virus (and the A/PR/8/34 NA noncoding regions, cytoplasmic tail domain, and transmembrane domain), which allowed us to measure NI titers without interference from anti-B HA antibodies (Fig. 2G). The NI titers determined with this virus were similar to the titers measured by ELISA, with no increases observed in sera from the prevaccination time point to the postvaccination time point (Fig. 2I). Again, we found good correlation between ELISA titers and H6NB_Yam88_ NI titers (r = 0. 8643, Fig. 2J).

### Prepandemic titers against the NA of the pandemic 2009 H1N1 virus are low.

**FIG 3:**
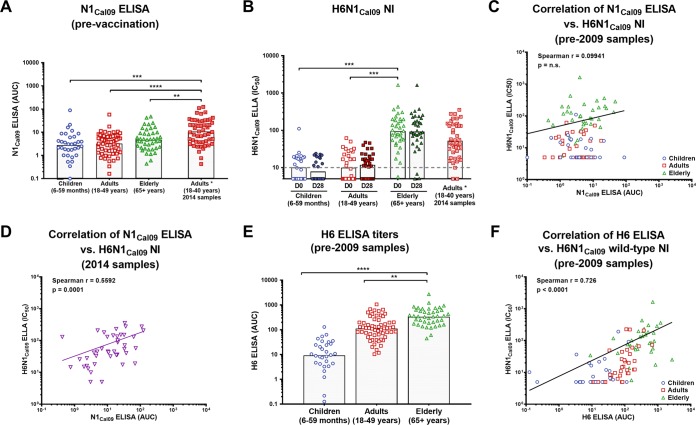
Binding and functionality of antibodies against the 2009 pandemic H1N1 NA. (A) Geometric mean Cal09 N1 ELISA titers (AUC) of sera from children, adults, and the elderly (prepandemic) and sera from adults collected after the 2009 pandemic. The postpandemic adult sera had significantly higher Cal09 N1 ELISA titers than the sera from the three age groups that were not exposed to the 2009 pandemic virus. (B) Mean ELLA titers against the H6N1_Cal09_ virus before and after vaccination. The dashed line indicates the starting serum dilutions used in the ELLA assays. (C) The Ella IC_50_ H6N1_Cal09_ values and the Cal09 N1 ELISA titers in sera from individuals not exposed to the 2009 pandemic H1N1 virus do not correlate (Spearman *r* = 0.09914). n.s., not significant. (D) Titers from postpandemic sera do correlate well (Spearman *r* = 0.5592, *P* = 0.0001). (E) Mean H6 HA ELISA titers of the prepandemic cohort (children, adults, and the elderly; collected on day 0). The elderly had high baseline titers compared to children and adults. (F) The ELLA IC_50_s for H6N1_Cal09_ correlate well with the ELISA titers measured against H6 for individuals not exposed to 2009 H1N1 pandemic virus (Spearman *r* = 0.726, *P* < 0.0001).

Subsequently, we assessed correlations between binding and NI for the N1 of the 2009 pandemic H1N1 virus. The serum samples were drawn from individuals in the three cohorts before 2009; thus, these individuals had therefore not yet been exposed to this virus. Not surprisingly, the ELISA titers measured with recombinant Cal09 N1 were very low in all age groups (Fig. 1A and Fig. 3A). Interestingly, the NI titers measured with H6N1_Cal09_ virus did not reflect the pattern seen with ELISA. While children and adults showed low titers as expected, the elderly cohort showed increased NI titers (Fig. 3B).

There was no significant correlation found between anti-NA ELISA titers and H6N1_Cal09_ NI titers (Fig. 3C). This suggests that the increased NI titers in the elderly were caused by interference from HA-specific antibodies. In an earlier study, we had investigated the prevalence of broadly protective anti-HA stalk antibodies in the same cohort and found higher anti-HA stalk titers in the elderly (30). These antibodies bind broadly to the conserved HA stalk and are also capable of binding to the H6 HA used in the H6NX viruses for the NIs. We therefore wondered if elevated titers of anti-HA stalk antibodies in the elderly might interfere with the NI assay in this cohort. We measured the reactivity of the sera to H6 HA and found an increase with age, with the elderly having the highest titers followed by the adults and with the children having the lowest titers (Fig. 3E). We correlated the anti-H6 titers with the H6N1_Cal09_ NI titers and indeed found a highly significant correlation (*r* = 0.726, Fig. 3F), indicating that anti-stalk/anti-H6 antibodies might contribute to the NI titers, specifically when low levels of NA-specific antibodies are present.

### Anti-HA stalk antibodies interfere with NI assays.

**FIG 4:**
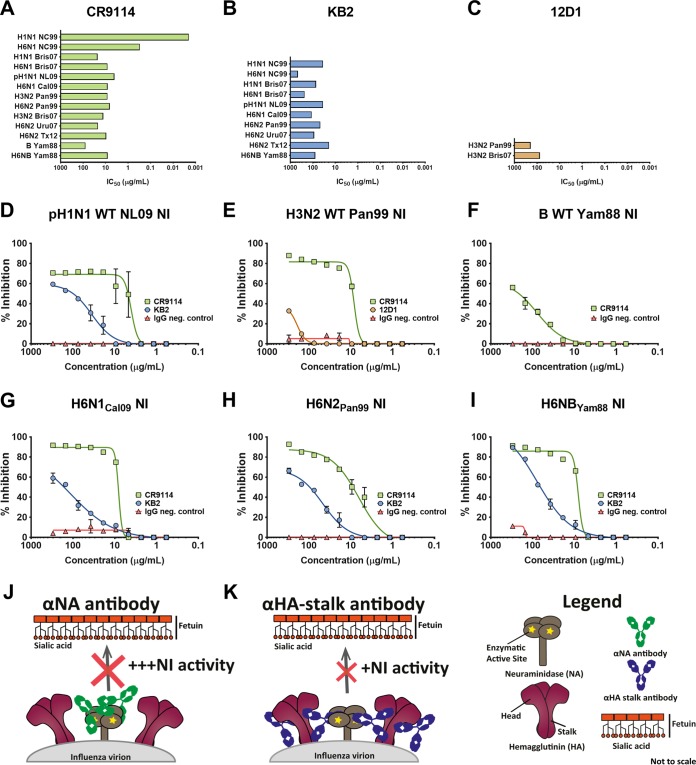
Anti-HA stalk antibodies interfere with NI activity in ELLA assays. (A) ELLA IC_50_ values for broadly reactive pan-HA stalk MAb CR9114 against H6NX and wild-type viruses. (B) ELLA IC_50_ values for broadly reactive group 1 MAb KB2 as measured with H6NX and wild-type viruses. (C) NI against H3N2 viruses in the presence of H3 stalk-reactive MAb 12D1. (D to I) Representative NI data from various H6NX and wild-type viruses in the presence of MAb CR9114, MAb KB2, or MAb 12D1 or an IgG negative-control MAb were plotted and fitted to nonlinear regression curves. (J and K) The data shown explain the potential mechanism of anti-HA stalk antibodies interfering with NI activity. (J) Anti-NA antibodies robustly interfere with neuraminidase activity by directly binding to NA and inhibition/shielding of the enzymatic site. (K) Anti-HA stalk antibodies bind to membrane-proximal sites on the HA and, via steric hindrance, indirectly shield the enzymatic site of NA and thus interfere with neuraminidase activity. However, this interference seems less robust than the inhibition from anti-NA antibodies.

The correlation analysis provided indirect evidence for interference of anti-HA stalk antibodies in the ELLA-based NI assay. To further investigate this phenomenon, we performed ELLAs with wild-type (wt) and H6NX viruses in the presence of anti-HA stalk monoclonal antibodies (MAbs). All three tested anti-stalk MAbs showed NI activity to various degrees. Human anti-stalk MAb CR9114 binds to all known influenza A virus HAs as well as to influenza B virus HA (34). Used in the ELLA assay, the MAb inhibited the NA activity of all wild-type and H6NX viruses (Fig. 4A and D to I). Similarly, anti-stalk MAb KB2 (35, 36) had activity against all H1- and H6-expressing viruses (Fig. 4B and D and G to I). Finally, we also tested anti-H3-stalk MAb 12D1 (37), which showed low activity (Fig. 4C and E). While anti-NA antibodies inhibit neuraminidase activity by direct binding to NA (Fig. 4J), the NI activity of anti-HA stalk antibodies is most likely caused by steric hindrance (Fig. 4K) as previously shown for anti-stalk MAb 9H10 and H10N8 viruses (16). In summary, anti-HA stalk MAbs contribute to inhibition of NA activity in assays performed with wild-type and H6NX viruses, depending on the MAb used.

## DISCUSSION

Immunity directed against the influenza virus NA has been shown to provide protection against viral infection in animal models (6, 12–23). Furthermore, several studies have suggested that NA-based immunity also correlates with protection from influenza virus infection and disease (7, 8, 14, 24). However, the breadth of anti-NA antibodies in the human population has not been extensively studied. Titers in adults and the elderly seemed to be highest against strains that these individuals had encountered early in life. As an example, the middle-aged cohort exhibited the highest N2 titer against Phil82 (H3N2), a virus that circulated when individuals in this cohort were younger than 15 years of age. Therefore, our data provide evidence for a phenomenon known as original antigenic sin, back-boosting, or antigenic imprinting which has been described in detail for influenza virus HA but not for the NA (27–29).

Interestingly, we found a striking difference between the titers against N1—which were relatively low—and the titers against N2 and influenza B virus NA, which were moderately high. This difference was detected with both the ELISA and the ELLA (NA inhibition), indicating that there might be an inherent difference in antigenicity between these NA subtypes. Reliance on this conclusion must be tempered by the fact that different substrates are used in the immunoassays involving N1 and N2. The higher titers for N2 may be a reflection of differences in stability between N1 and N2 NA. For the virus-based NI assay, the NA content of the virion might play a role as well, although a study by Tanimoto and colleagues did not find statistically significant differences in NA content among H1N1, H3N2, and influenza B viruses (38).

Adults and the elderly have high titers of anti-influenza B virus NA antibodies, while children are basically nonreactive, likely because they have not been exposed to influenza B virus. Increasing the influenza B virus NA titers in children by vaccination could improve protection against this infection (39).

When we examined the relationship between binding (ELISA) and functional (ELLA) antibodies, we found a good correlation for prepandemic seasonal N1, N2, and type influenza B virus NA. However, pH1N1 ELISA titers against N1, which were very low, did not correlate with NI titers. Here we present preliminary evidence suggesting that anti-HA stalk antibodies, which also bind to H6 HA of the reassortant viruses used in the ELLA, could be the cause of the low agreement between the two assays. So far, this phenomenon has been described only for H10N8 viruses and anti-stalk MAb 9H10 (16, 40). When three anti-HA stalk monoclonal antibodies were tested in NI assays with wild-type and H6NX viruses, all three showed activity to various degrees. This needs to be considered when NI activity is measured using the ELLA in sera from individuals who have HA stalk-specific antibodies.

In summary, we have assessed the breadth, functionality, and isotype/subtype usage of anti-NA antibodies in children, adults, and the elderly. Anti-NA titers increase with age, but the magnitude of the response is subtype dependent and appears to be low for N1 whereas it is higher and cross-reactive for N2 and influenza B virus NA. In addition, we provide evidence for original antigenic sin of the influenza virus neuraminidase based on the finding that the titers are always highest against the NAs of the viruses to which the subjects were first exposed. It will be interesting to determine in detail in future studies how anti-NA titers are shaped by natural infection and vaccination and how adjuvants would influence vaccine-induced anti-NA immunity. Understanding the characteristics of the NA-specific response will help researchers to design vaccines that induce high and cross-reactive anti-NA antibody titers in all age groups and against all subtypes.

## MATERIALS AND METHODS

### Cells and viruses.

Madin-Darby canine kidney (MDCK) cells were grown in complete Dulbecco’s modified Eagle’s medium (DMEM; Life Technologies, Inc.). DMEM was supplemented with 10% fetal bovine serum (FBS; HyClone, Sigma-Aldrich) and a mixture of antibiotics (penicillin [100 U/ml] and streptomycin [100 µg/ml] [Pen-Strep]; Gibco). Sf9 insect cells were grown in TNM-FH insect cell medium (Gemini Bioproducts) supplemented with 10% FBS and Pen-Strep antibiotics. BTI-TN-5B1-4 (High Five) cells (Vienna Institute of Biotechnology subclone [41]) were grown in serum-free SFX medium (HyClone) supplemented with Pen-Strep. Viruses used in this study include wild-type (wt) strains pH1N1 (A/Netherlands/04/2009 [NL09]—antigenically equivalent to Cal09), H1N1 (A/New Caledonia/20/1999 [NC99]), H3N2 (HA and NA from A/Panama/2007/1999 [Pan99] and internal genes from A/Puerto Rico/8/34 [PR8, H1N1]), and B (B/Yamagata/16/1988 [Yam88]) and reassortant strains H6N1_Cal09_ (HA from A/turkey/Massachusetts/3740/1965 [Mass65, H6N2], NA from A/California/04/2009 [Cal09, pH1N1], and internal genes from PR8), H6N1_NC99_ (HA from Mass65, NA from NC99, and internal genes from PR8), H6N2_Pan99_ (HA from Mass65, NA from Pan99, and internal genes from PR8), H6N2_Uru07_ (HA from Mass65, NA from A/Uruguay/716/2007, and internal genes from PR8), H6N2_Tx12_ (HA from Mass65, NA from A/Texas/50/2012, and internal genes from PR8), and H6NB_Yam88_ (HA from A/mallard/Sweden/82/2002 [H6N1], NA from B Yam88, and internal genes from PR8).

The novel H6NB_Yam88_ virus was rescued through reverse genetics by use of an eight-plasmid system. The NA segment was generated by using In-Fusion cloning (Clontech). The packaging signals (noncoding regions, cytoplasmic tail domain, and transmembrane domain) for this clone were derived from PR8 N1, while the ectodomain used was from Yam88 NB. The HA used was derived from A/mallard/Sweden/82/2002, and the other six segments (PB2, PB1, PA, NP, M, and NS) were derived from PR8. All segments were cloned into a pDZ vector, a bidirectional transcription plasmid that utilizes polymerase (Pol) I and Pol II promoters. 293T cells were transfected with 500 ng of plasmids for each influenza virus segment using Lipofectamine 2000 (Thermo Fisher Scientific). After 24 h, cells and supernatant were collected and injected into 8-day-old embryonated chicken eggs. Viral rescue cultures were initially screened by performing a hemagglutination assay. The resulting virus was plaque purified and expanded in embryonated chicken eggs.

The viruses described above were grown in 8-to-10-day-old embryonated chicken eggs (Charles River Laboratories, Inc.). Inoculated eggs with wild-type influenza A and reassortant viruses were incubated at 37°C for 48 h, and wt influenza B viruses were incubated at 33°C for 72 h. Eggs were then cooled to 4°C for 4 to 12 h, harvested, and clarified by low-speed centrifugation (3,000 rpm, 20 min). Virus titers were determined by plaque assay on MDCK cells in the presence of tosyl phenylalanyl chloromethyl ketone (TPCK)-treated trypsin.

### Recombinant HA and NA proteins.

All recombinant proteins were expressed in BTI-TN-5B1-4 cells and purified from cell culture supernatant as previously described (6, 42, 43). Recombinant NA and HA proteins used in this study were derived from the following isolates: A/South Carolina/1/1918 (N1), A/USSR/90/1977 (N1), A/New Caledonia/20/1999 (N1), A/Vietnam/1203/2004 (N1), A/California/04/2009 (N1), A/Singapore/1/1957 (N2), A/Hong Kong/1/1968 (N2), A/Philippines/2/1982 (N2), A/chicken/Hong Kong/G9/1997 (N2), A/Panama/2007/1999 (N2), A/Hong Kong/4801/2014 (N2), B/Lee/1940 B (NA), B/Yamagata/16/1988 (NB), B/Malaysia/2506/2004 (Mal04, NB), B/Florida/4/2006 (Flor06, NB), B/Wisconsin/01/2010 (Wisc10, NB), and A/mallard/Sweden/82/2002 (H6). Briefly, recombinant baculoviruses expressing soluble proteins were propagated in Sf9 insect cells. The recombinant baculoviruses were used to infect BTI-TN-5B1-4 cells at a multiplicity of infection of 10 to express secreted soluble recombinant proteins that were then purified from cell culture supernatants using nickel-nitrilotriacetic acid (Ni-NTA) resin (Qiagen) as described previously (42).

### Human serum samples.

Human serum samples were obtained from clinical trials performed by the Protein Sciences Corporation. The trials were randomized, double-blind, multicentered trials determining the safety, reactogenicity, and immunogenicity of Flublok, a recombinant influenza virus vaccine. All participants in the trial received 135 µg of recombinant HA (45 µg of A/New Caledonia/20/1999-like H1, 45 µg of A/Wisconsin/67/2005-like H3, and 45 µg of B/Ohio/01/2005-like B HA). The trials were performed in three different cohorts: children (6 to 59 months of age), adults (18 to 49 years of age), and elderly (≥65 years of age). Human serum sample collection was completed between May 2006 and May 2008. Further information regarding the clinical trials can be found on ClinicalTrials.gov with the following identifiers: NCT00336453 (children [44]), NCT00539981 (adults [45]), and NCT00395174 (elderly [46]). Serum samples were obtained prevaccination (day 0) and postvaccination (day 28) based on sample availability for both time points and were then subsequently analyzed at the Icahn School of Medicine at Mount Sinai, New York, NY (protocol/exempt determination number 15-00126). Commercially available postpandemic 2009 serum samples (Innovative Research), collected in 2014, with no identifying information other than age, sex, and race were also used in this study.

### Enzyme-linked immunosorbent assay (ELISA).

Microtiter 96-well plates (Immulon 4 HBX; Thermo Fisher Scientific) were coated with 2 µg/ml HA or NA protein (50 µl/well) diluted in coating solution (KPL) and were then incubated overnight at 4°C. The following day, plates were washed three times with phosphate-buffered saline (PBS) containing 0.1% Tween 20 (PBS-T) and then blocked with PBS-T containing 3% goat serum (Life Technologies, Inc.) and 0.5% milk powder (blocking solution). Blocking solution was discarded after 1 h of incubation at 20°C. Serum samples diluted to a starting concentration of 1:100 were first added to the plates and then serially diluted 1:2 in blocking solution. The final volume in all wells after dilution was 100 µl. After a 2-h incubation period at 20°C, plates were washed 3 times with PBS-T. Secondary antibody (50 µl/well) diluted in blocking solution was added, and the plates were incubated for 1 h at 20°C and washed four times with PBS-T. After developing for 10 min with SigmaFast *o-*phenylenediamine dihydrochloride (OPD; Sigma), the reaction was stopped with 3 M hydrochloric acid. The plates were immediately read at an optical density (OD) of 490 nm using a Synergy H1 hybrid multimode microplate reader (BioTek). The background of each plate (and 3 standard deviations) was calculated for each plate and used as the lower limit for area-under-the-curve analysis using Prism 7 (GraphPad). The following secondary antibodies were used: anti-human IgG (Fab-specific) horseradish peroxidase (HRP) antibody (Sigma A0293) (1:3,000), anti-human IgA (α-chain-specific) HRP antibody (Sigma A0295) (1:3,000), anti-human IgM (μ-chain-specific) HRP antibody (Sigma A6907) (1:3,000), anti-human IgG1 Fc-HRP (Southern Biotech 9054-05) (1:3,000), anti-human IgG2 Fc-HRP (Southern Biotech 9060-05) (1:3,000), anti-human IgG3hinge-HRP (Southern Biotech 9210-05) (1:3,000), and anti-human IgG4 Fc-HRP (Southern Biotech 9200-05) (1:10,000). All serum samples were tested using anti-human IgG (Fab-specific) HRP antibody. To find differences in antibody subtype/isotype specificity (IgG1, IgG2, IgG3, IgG4, IgA, IgM), 20 individuals of each age group (day 0; prevaccination) were randomly selected and tested for all IgG subtypes, IgM, and IgA using NC99 N1, Pan99 N2, and B Yam88 NA substrates.

### Receptor-destroying enzyme (RDE) treatment.

Human serum samples were treated with receptor-destroying enzyme (RDE) (Denka Seiken) to remove nonspecific/background NA inhibition. A 25-μl volume of human serum was added to 75 µl of RDE. The mixture was incubated at 37°C for 16 to 18 h. RDE was inactivated with 75 µl of 2.5% sodium citrate and then heat inactivated at 57°C for 30 min. Finally, 75 µl of PBS was added to give a final human serum sample concentration of 1:10.

### Enzyme-linked lectin assay (ELLA) to determine neuraminidase inhibition (NI).

The ELLA assay was performed according to previously published protocols (25, 26). First, a neuraminidase assay (NA) was performed to determine the optimal virus concentration to be used in the NI assay. The NA assay was performed for all virus stocks. Microtiter 96-well plates (Immulon 4 HBX; Thermo Fisher Scientific) were coated with 25 µg/ml (100 µl/well) of fetuin (Sigma) diluted in coating solution (KPL) and incubated overnight at 4°C. The following day, virus stocks were serially diluted 1:2 in PBS containing 5% bovine serum albumin (blocking solution) in a separate 96-well plate, starting with the undiluted stock. The final volume in all wells after dilution was 150 µl. The plates were incubated at room temperature for 1.5 h. During the incubation time, the fetuin-coated plates from the previous day were washed three times with PBS containing 0.1% Tween 20 (PBS-T) and then blocked with blocking solution for 1 h at room temperature. After blocking was performed, the fetuin-coated plates were again washed three times with PBS-T. Viral dilutions (100 µl) were then transferred to the fetuin-coated plates and incubated at 37°C for 2 h. The plates were washed four times with PBS-T. Peanut agglutinin conjugated to HRP (PNA-HRP; Sigma) at a concentration of 5 µg/ml diluted in PBS (100 µl/well) was added, and the reaction mixture was incubated in the dark at room temperate for 1.5 h. After incubation, the plates were washed again four times with PBS-T and developed for 5 min with SigmaFast OPD (Sigma). The developing process was stopped with 3 M hydrochloric acid, and the reaction was immediately read at an optical density (OD) of 490 nm using a Synergy H1 hybrid multimode microplate reader (BioTek). To determine the optimal virus concentration to be used in the NI assay, the absorbance data were plotted in GraphPad Prism 7. The data were fitted to a nonlinear regression curve to determine the 50% effective concentration (EC_50_) and the 90% effective concentration (EC_90_). The EC_90_ was subsequently used for NI assays.

For NI assays, microtiter 96-well plates (Immulon 4 HBX; Thermo Fisher Scientific) were coated using a method similar to that employed for the NA assay. The next day, RDE-treated human serum samples were serially diluted 1:2 in separate 96-well plates, with a starting concentration of 1:10. The final volume of diluted serum samples in all wells was 75 µl. Virus stocks were diluted in PBS to the determined EC_90_ concentration. After virus (75 µl/well) was added to the serum plates, the plates were incubated at room temperature for 1.5 h. The fetuin-coated plates were blocked and then washed under conditions similar to those used for the NA assay. A 100-µl volume of the virus/serum mixture was transferred to the washed fetuin-coated plates, which were then incubated for 2 h at 37°C. The plates were washed four times with PBS-T, and PNA-HRP (Sigma) diluted to 5 µg/ml in PBS (100 µl/well) was added. The NA assay protocol was followed for the remaining NI assay steps. The human serum sample reactivity was determined by subtracting the background absorbance value from the raw absorbance value of human serum samples. The obtained value was divided by the average value from virus-only control wells and then multiplied by a factor of 100 to calculate the NA activity. Percent NI was determined by subtracting NA activity from 100%. Using GraphPad Prism 7, the percent NI was fitted to a nonlinear regression to determine the 50% inhibitory concentration (IC_50_) of the serum samples.

### Statistical analysis.

GraphPad Prism 7 was used to perform all statistical analyses. The three different age groups were compared using one-way analysis of variance with Tukey multiple-comparison tests. A Spearman correlation test was used for all correlation analyses. ELISA and ELLA data are shown as geometric means. Statistical significance is indicated as follows: n.s. (not significant), *P* > 0.05; *, *P* ≤ 0.05; **, *P* ≤ 0.01; ***, *P* ≤ 0.001; ****, *P* ≤ 0.0001. The phylogenetic trees used in this study were built using ClustalOmega, and data were visualized using FigTree. The amino sequences for the NA proteins were obtained using GenBank and the Global Initiative on Sharing All Influenza Data (GISAID).
