# Supplementary figures and images for: Analysis of Anti-Influenza Virus Neuraminidase Antibodies in Children, Adults, and the Elderly by ELISA and Enzyme Inhibition: Evidence for Original Antigenic Sin
Source: mBio. 2017 Mar 21;8(2):e02281-16. doi: 10.1128/mBio.02281-16 (PMC5362038; doi:10.1128/mBio.02281-16)

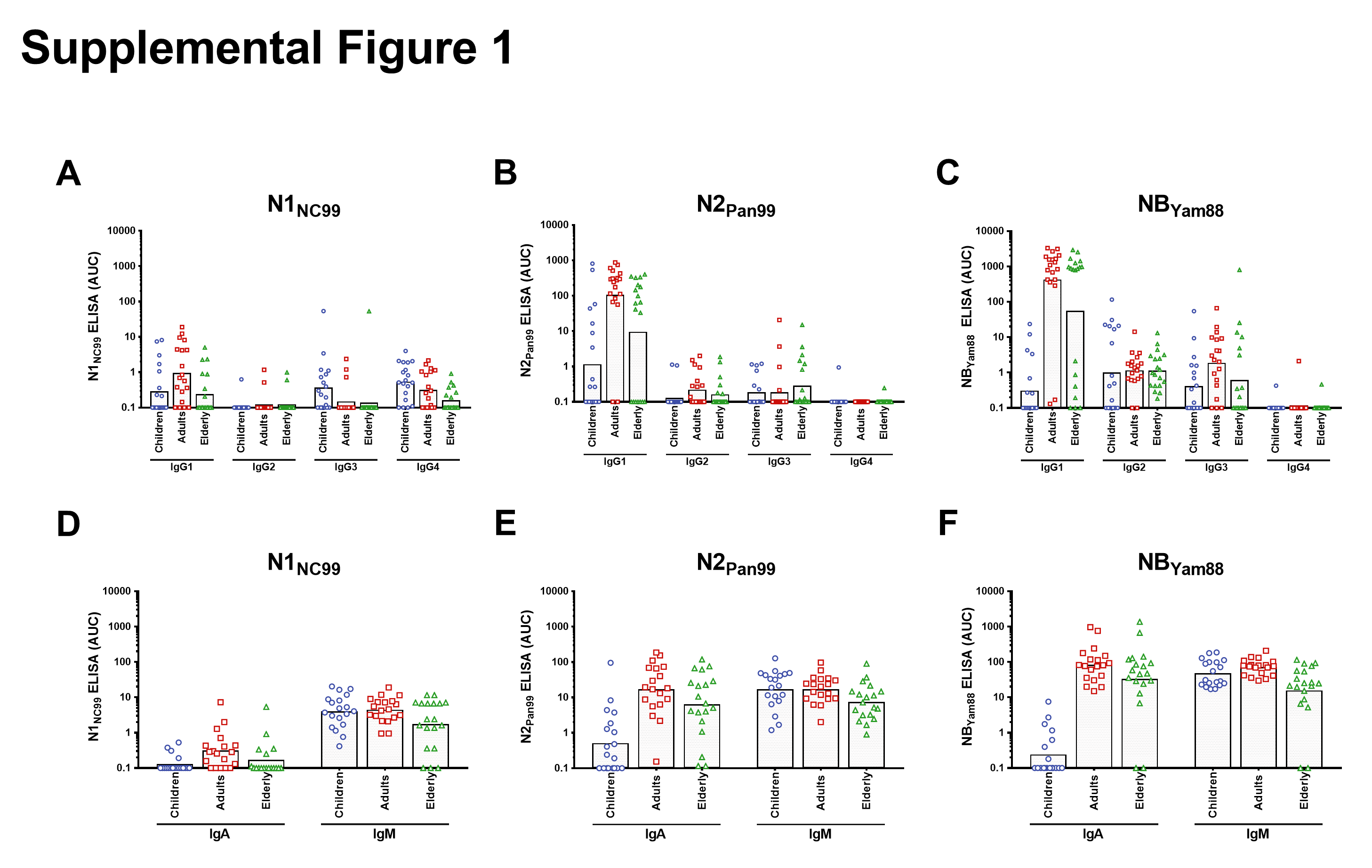

Supplement: FIG S1 [file mbo002173244sf1.tif]
